# CBA: Cluster-Guided Batch Alignment for Single Cell RNA-seq

**DOI:** 10.3389/fgene.2021.644211

**Published:** 2021-04-13

**Authors:** Wenbo Yu, Ahmed Mahfouz, Marcel J. T. Reinders

**Affiliations:** ^1^Department of Control Science and Engineering, Harbin Institute of Technology, Harbin, China; ^2^Delft Bioinformatics Lab, Delft University of Technology, Delft, Netherlands; ^3^Leiden Computational Biology Center, Leiden University Medical Center, Leiden, Netherlands; ^4^Department of Human Genetics, Leiden University Medical Center, Leiden, Netherlands

**Keywords:** batch correction, auto-encoder, single-cell RNA sequencing, clustering, data integration

## Abstract

The power of single-cell RNA sequencing (scRNA-seq) in detecting cell heterogeneity or developmental process is becoming more and more evident every day. The granularity of this knowledge is further propelled when combining two batches of scRNA-seq into a single large dataset. This strategy is however hampered by technical differences between these batches. Typically, these batch effects are resolved by matching similar cells across the different batches. Current approaches, however, do not take into account that we can constrain this matching further as cells can also be matched on their cell type identity. We use an auto-encoder to embed two batches in the same space such that cells are matched. To accomplish this, we use a loss function that preserves: (1) cell-cell distances within each of the two batches, as well as (2) cell-cell distances between two batches when the cells are of the same cell-type. The cell-type guidance is unsupervised, i.e., a cell-type is defined as a cluster in the original batch. We evaluated the performance of our cluster-guided batch alignment (CBA) using pancreas and mouse cell atlas datasets, against six state-of-the-art single cell alignment methods: Seurat v3, BBKNN, Scanorama, Harmony, LIGER, and BERMUDA. Compared to other approaches, CBA preserves the cluster separation in the original datasets while still being able to align the two datasets. We confirm that this separation is biologically meaningful by identifying relevant differential expression of genes for these preserved clusters.

## 1. Introduction

Single-cell RNA sequencing (scRNA-seq) technologies are important to study the cellular heterogeneity in biological tissues (Hie et al., 2018; Svensson et al., 2018; Lin et al., 2019). Compared with bulk RNA sequencing, scRNA-seq aims at detecting cellular differences in seemingly homogeneous populations (Butler et al., 2018; Büttner et al., 2019). By analyzing cellular characteristics via their high-throughput gene expression profiles, scRNA-seq increases the resolution of cell type differences tremendously. A plethora of platforms and techniques are available, but they all suffer from technical biases (such as RNA capture and reverse transcription efficiency) (Lopez et al., 2018; Schuyler et al., 2019; Wang et al., 2019). Consequently, these batch effects challenge the integration of scRNA-seq datasets, which is often necessary to compare biological conditions (Haghverdi et al., 2018; Li and Li, 2018).

Typically, the quantification of these batch effects is clouded by biological differences between batches, such as additional cell types or cells in different states (Tran et al., 2020). When trying to correct for batch effects, these biological differences should be preserved while differences caused by technical effects should be removed. Existing integration techniques for scRNA-seq that try to resolve these batch effects have been divided into three categories (Chazarra-Gil et al., 2021), based on where the initial alignment takes place: either (1) in the original high dimensional space (*Seurat v3, mnnCorrect*, and *Scanorama*), (2) the final projected space (*Harmony* and fastMNN), or (3) an in-between space (*BBKNN*). For example, *Seurat v3* (Stuart et al., 2019) uses diagonalized canonical correlation analysis (CCA) to align directions of variations guided by “anchors” in the reference patch. “Anchors” are detected by mutual nearest neighbors (MNN) pairs between the query batch and the reference batch and they represent single cells in a shared biological state. *mnnCorrect* (Haghverdi et al., 2018) also uses MNN to match cell types and consequently learns biological corrections for all pairs to merge the batches. *Scanorama* (Hie et al., 2019) detects cell pairs using MNN after data compression by singular value decomposition (SVD) and an approximate NN search to reduce the nearest neighbor query time. *Harmony* (Korsunsky et al., 2019) and fastMNN (Haghverdi et al., 2018) first generate a low dimensional embedding of the original count matrices, and then aligns the data in this space, whereas *BBKNN* (Polanski et al., 2020) uses MNN to detect neighbors in a reduced PCA space and outputs neighbor graphs finally. All these existing techniques have in common that they are fully unsupervised, i.e., the alignments are not guided by information on cell types. These methods perform well; however, they do not take into account the cellular heterogeneity as present in single cell data sets. Some deep learning techniques tend to simulate this heterogeneity when training the alignment networks. DESC (Li et al., 2020) constructs non-linear mapping functions through iterative self-learning and aligns cells using a deep neural network, trying to move each single cell closer to its nearest cluster. BERMUDA (Wang et al., 2019) uses an autoencoder to align cells and identifies similar clusters across batches to retain cluster similarity. A loss function based on maximum mean discrepancy (MMD) is used to optimize the resulting alignment and retain the cellular homogeneity and heterogeneity.

In this work, we propose a cluster-driven batch alignment (CBA) framework for scRNA-seq datasets, to merge cells from the same population across batches while retaining their unique biological signals. CBA uses a deep learning model which incorporates a pre-clustering step to preserve the structure of each individual dataset. To retain these structures as well as align the batches, we incorporate intra-batch and the inter-batch similarities into the loss function used for training our deep learning model. The intra-batch similarity loss aims to keep cells from the same batch close to each other when they are from the same clustered group. The inter-batch similarity loss minimizes the distance between cells from two matched clusters across batches. In addition, we exploit a cellular reconstruction loss aimed to reconstruct the original cellular expression profiles (the auto-encoder part) and a classification loss which predicts whether two cells within one batch are from the same cluster. Our results illustrate that CBA is not only able to integrate cells from different batches, but also preserves the existing heterogeneous cellular biological signals within each batch. We show that these batch-specific signals correspond to subtle but meaningful diversities of cells within the same cluster.

## 2. Materials and Methods

### 2.1. Datasets

We used two pairs of datasets to evaluate our method (Table 1). First, we used human pancreatic islet datasets measured by different technologies, and downloaded from the Seurat package^1^. We extracted one smartseq2 dataset (E-MTAB-5061) and one celseq2 dataset (GSE85241). Second, we used Mouse Cell Atlas (MCA) datasets^2^ (Ha et al., 2018) which contains cells from 37 organs and the fluorescence activated cell sorting (FACS) datasets from Tabula Muris^3^ (Tabula, 2018). We selected the mouse lung datasets from MCA and the Tabula Muris which were measured using two different technologies, Microwell-seq for MCA and SMART-Seq2 for the FACS-sorted cells from Tabula Muris. In the remaining of the manuscript, we refer to the Mouse Cell Atlas data as MCA and the Tabula Muris data as TM.

**Table 1:** Cell type information of the utilized scRNA-seq datasets.

**Dataset**	**Batch**	**Cells**	**Populations**	**Filtered genes**	**Highly variable genes number**
Pancreas	smartseq 2	2,317	8	16,999	2,923
	celseq 2	2,158	8	16,999	2,923
Mouse lung	MCA	809	5	8,264	1,821
	TM	1,283	5	8,264	1,821

For all datasets, we used the same data preprocessing using the *Scanpy* package in Python. Considering that clusters with too few cells will influence the training process of the integration module, we followed (Wang et al., 2019) and removed cells that belong to some clusters that have <10 cells (here we used the cluster annotation provided in the original publication). After that, genes expressed in too few cells (50 cells for pancreas datasets and 100 cells for mouse lung datasets) were removed and highly variable genes were selected (*scanpy.pp.highly_variable_genes()*, default parameters). Table 1 gives an overview of the retained data for each dataset. After the gene filtering, gene counts are normalized by the total count of all genes within a cell and log transformed [using *scanpy.pp.normalize_per_cell()* and *scanpy.pp.log1p()*, where *counts*_*per*_*cell*_*after* is set as 10^4^]: ${\tilde{G}}_{i,j}=\log\left(\left(\frac{G_{i,j}}{\underset{i}{\sum }G_{i,j}}\times 10^{4}\right)+1\right)$, where ${\tilde{G}}_{i,j}$ is the normalized expression level of gene *i* in cell *j*. Next, we retained the top-*k* principal components for the downstream analysis in which *k* was decided on the bending point of the variance explained per component (evaluating *k* ∈ [10, 15, 20, 25, 30]).

### 2.2. Cluster-Driven Batch Alignment (CBA)

**Figure 1:**
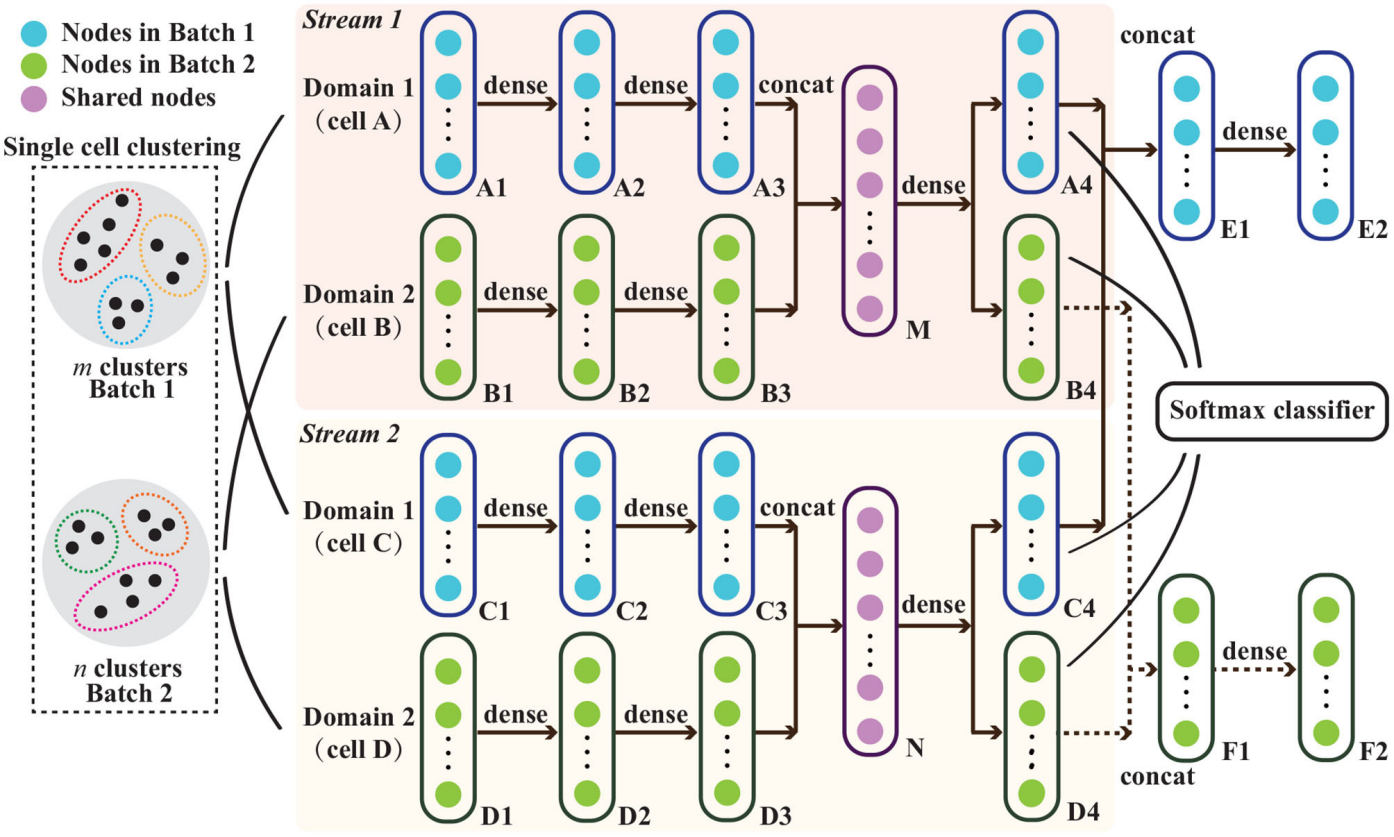
Schematic representation of the proposed cluster-driven batch alignment (CBA) method to align single cell RNA-seq measured in two different batches. The unsupervised clustering for cells from both batches and the network architecture in CBA are shown and the explanation of various nodes are listed on the top left corner. Cell A and cell C are from batch one, and cells B and D are from batch two. At its core, the alignment is done using an autoencoder where cells A&B are aligned and embedded in a lower dimensional representation M and, simultaneously, cells C&D are aligned and embedded in N. M&N are subsequently used to represent the aligned cells, e.g., to make a UMAP visualization. Details on the autoencoder as well as the classification layer can be found in the section that describes CBA.

The complete CBA workflow is shown in Figure 1. The main idea is that we aim to retain the data structure in the separate batches as much as possible. Hereto, we first cluster the data in each batch to capture this structure. Then we match the clusters between the two batches and use this matching as guidance for the data integration, i.e., distances between cells in matched clusters should be small at the expense of distances between cells belonging to non-matched clusters. We then use an autoencoder to embed the data of both batches into a lower dimensional space to achieve the alignment between the batches with the constraints that: (1) the reconstructed expression is similar to the original expression (reconstruction loss), (2) the distances between cells of the same batch that belong to the same cluster are kept small (structure preserving loss), (3) the distances between cells of matching clusters is kept small (cluster preserving loss), and (4) focusing on features that are informative for determining whether clusters are matching or not (cluster prediction loss). The following discusses each of these steps in more detail.

First, we cluster the cells in each batch separately to capture the cell identities. Hereto, we applied the Louvain clustering algorithm (Levine et al., 2015). We use the *Scanpy* implementation, and the resolution parameter (related to the number of clusters, and selected based on UMAP visualizations) is set to 0.5 for the mouse lung datasets, the default value for the pancreas datasets, and to 0.25 for the pancreas datasets in which we removed one of the cell types (*alpha* cells from batch 1). Next, we create a cluster matrix in which an element equals one when two cells (*p* and *q*) from the same batch *b* belong to the same cluster, $C_{pq}^{b}=1$, and zero otherwise. To match the resulting clusters between the two batches, we calculate the pairwise distances between cells of cluster *i* in batch one with cells of cluster *j* in batch two:

(1) $$\begin{array}{r} U_{i,j}=log_{10}\left\{\frac{1}{k_{i}^{1}*k_{j}^{2}}\overset{k_{i}^{1}}{\underset{p\text{=}1}{\sum }}\overset{k_{j}^{2}}{\underset{q\text{=}1}{\sum }}d_{cos}\left(c_{p}^{1},c_{q}^{2}\right)\right\} \end{array}$$

where $k_{i}^{1}$ and $k_{j}^{2}$ are the number of cells in clusters *i* and *j* of batch one and two, respectively, and *U*_*i, j*_ has size *K*^1^ × *K*^2^, with *K*^1^ and *K*^2^ equaling the number of clusters in batch one and two, respectively. The log10 is used to emphasize small distances. As distance between cells, we used the cosine similarity, representing the angular similarity between the two gene expression vectors: $d_{cos}\left(c_{p}^{1},c_{q}^{2}\right)=1-\frac{\sum \left(c_{p}^{1}\text{×}c_{q}^{2}\right)}{||c_{p}^{1}||\text{×}||c_{q}^{2}||}$. Next, we match a cluster *i* of batch one with the most similar cluster in batch two, i.e., cluster j^*^ which has the minimum *U*_*ij*_ over all *j*. With that we create a matrix *M*^1^, which indicates for every pair of cells ($c_{p}^{1}$ and $c_{q}^{2}$), from batch one and two, whether they are originating from matching clusters ($M1_{pq}=||\text{− }U_{ij^{*}})||$) or not (*M*1_*pq*_ = 0), where ||·|| represents a 0–1 normalization. We also create a similar matrix *M*2, which indicates the Euclidean distance between cells. The final matrix *M*, *M*_*pq*_ = *M*1_*pq*_ × *M*2_*pq*_, indicates the distance between cells in different batches when belonging to matched clusters (*M*_*pq*_ > 0) or specifies that the cells belong to non-matching clusters (*M*_*pq*_ = 0) indicating that distances between these cells do not have to be preserved.

To integrate cells from two batches, we use an autoencoder. The network architecture is shown in Figure 1. Inputs are the cells of the different batches (expression vectors related to the selected PCs), the clustering of the separate batches, as well as their matching. At the core of the autoencoder is the embedding of a cell to a lower dimensional representation in such a way that the expression profile can be reconstructed as good as possible, i.e., the reconstruction loss, L_*r*_ should be minimized. In Figure 1 this can be seen by following cell A: the initial representation A1 is embedded with stacked dense layers to a lower presentation A3, which is then reconstructed to the original dimensions A4. To accomplish an alignment between the two batches, we perform this auto-encoding for two cells from the two different batches simultaneously. Moreover, we do that for two pairs of cells at the same time (for reasons explained later) in different streams, i.e., pair A&B and pair C&D in stream 1 and 2, respectively (Figure 1). Focusing on pair A&B, their representation is concatenated and embedded in M and reconstructed in A4 and B4. The reconstruction loss then requires that E2 is similar to the concatenated representation of A&C. For both pairs this becomes:

(2) $$\begin{array}{r} L_{r}=\frac{1}{2\epsilon_{r}}\sum {\left(\left(A1||C1\right)-E2\right)}^{2}+\frac{1}{2\epsilon_{r}}\sum {\left(\left(B1||D1\right)-F2\right)}^{2} \end{array}$$

where *x*||*y* represent the concatenation of *x* and *y*, and *ϵ*_*r*_ is the number of features in A1/B1/C1/D1.

To enforce the preservation of the local structure of the data, we require, in addition, that cells from the same cluster (in one batch) should be kept close to each other after the embedding, which we express by a structure preserving loss L_*s*_. For this reason, we make use of the two streams. Then, requirements on cells from the same batch, in Figure 1 A&C as well as B&D, can be formulated. Focusing on A&C, we require that the reconstructed versions A4&C4 are close to each other when they are from the same cluster, and we do not put any requirement when they are from different clusters. For both pairs this structure preserving loss becomes:

(3) $$\begin{array}{r} L_{s}=\frac{1}{\epsilon_{s}}\sum {\left(A4-C4\right)}^{2}C_{A4,C4}^{1}+\frac{1}{\epsilon_{s}}\sum {\left(B4-D4\right)}^{2}C_{B4,D4}^{2} \end{array}$$

where $C_{pq}^{b}=1$ when two cells belong to the same cluster within batch *b* and zero otherwise. *ϵ*_*s*_ is the number of features in A4/B4/C4/D4.

Similarly, we require that two cells from different batches but matching clusters also should be close together. For this cluster preserving loss, L_*c*_, we thus put requirements on pairs A&B and C&D in Figure 1. Focusing on A&B, we require that their embedded representations A4&B4 are close to each other when they are in matching clusters and there are no constraints when they are not in matching clusters. For both pairs this then becomes:

(4) $$\begin{array}{r} L_{c}=\frac{1}{\epsilon_{c}}\sum {\left(A4-B4\right)}^{2}M_{A4,B4}+\frac{1}{\epsilon_{c}}\sum {\left(C4-D4\right)}^{2}M_{C4,D4} \end{array}$$

where *M*_*pq*_ > 0 when cell *p* from batch 1 and cell *q* from batch 2 belong to matching clusters and zero otherwise. *ϵ*_*c*_ is the number of features in A4/B4/C4/D4. Note that when cells belong to matching clusters, the importance of putting them close to each other is increasing with their original similarity, as defined by *M*_*pq*_.

In addition to the reconstruction losses, we also direct the embedding toward those features that are informative for determining whether clusters are matching or not. Hence, when presented with pairs of cells from different batches (A&C or B&D), we build a classifier based on their reconstructed data (A4&C4 and B4&D4) using a softmax classifier, such that the classifier outputs the predicted cluster label, trying to fit the one-hot cluster label. The performance of the classifier is recapitulated into a cluster prediction loss, L_*p*_:

(5) $$\begin{array}{l} \begin{array}{c} L_{p}=\frac{1}{\epsilon_{p}}\sum {\left(softmax\left(A4\right)-\text{Ω}\left(A4\right)\right)}^{2}\\ \;+\frac{1}{\epsilon_{p}}\sum {\left(softmax\left(B4\right)-\text{Ω}\left(B4\right)\right)}^{2}\\ \;+\frac{1}{\epsilon_{p}}\sum {\left(softmax\left(C4\right)-\text{Ω}\left(C4\right)\right)}^{2}\\ \;+\frac{1}{\epsilon_{p}}\sum {\left(softmax\left(D4\right)-\text{Ω}\left(D4\right)\right)}^{2} \end{array} \end{array}$$

where Ω(*x*) represents the one-hot clustered label of *x* and *ϵ*_*p*_ is the number of features in A4/B4/C4/D4. The final loss function is then defined as the sum of the individual loss functions:

(6) $$\begin{array}{r} L_{c}=L_{r}+L_{s}+L_{c}+L_{p} \end{array}$$

The autoencoder is trained using the Adam optimizer with a learning rate set to 5 × 10^−4^ and training is stopped when all losses converge stably (their values do not decrease over a period of time).

### 2.3. Experimental Settings

Experiments were conducted using Python 3.7.0 (Spyder) and R 4.0.2 on a PC with Intel Core i7-6700 CPU and 8-GB RAM. For CBA, the training time per epoch was ~1 s. Less than 10,000 epochs are needed for training per experiment (early stopping is used to prevent overfitting). The used memory vs. the training time is shown in Supplementary Figure 1 (about 1.18 GB), the memory is queried using the *psutil* module in Python. In LIGER, the parameter *k* in *optimizeALS*() is set to 20 for the pancreas datasets and to 40 for the mouse lung datasets (as recommended by the authors). For BERMUDA an important parameter is the threshold which is advised to be chosen between 0.85 and 0.90, after visual exploration we set this value to 0.85. For the other alignment methods, the preprocessing modules (including normalization and log transformation) and codes are consistent with their papers and GitHub homepages.

### 2.4. Evaluation Metrics

We used several metrics to quantitatively evaluate the alignment of two datasets. kBET (Büttner et al., 2019) measures how well the datasets are mixed by exploring the confusion matrix in neighborhood of cells. The silhouette score (SC) was used to assess how well the data can be clustered. The adjusted rand index (ARI) was used to compare how well a clustering of the aligned dataset agrees with the clusterings in each of the dataset separately. The normalized mutual information (NMI) also captures the similarity between the above clusterings by the normalization of the mutual information score. Finally, we use the Fowlkes-Mallows index (FMI) to quantify the clustering consistency by the positive predictive rate (precision) and the true positive rate (recall).

## 3. Results

### 3.1. CBA Resolves Batch Effects Across scRNA-seq Datasets

**Figure 2:**
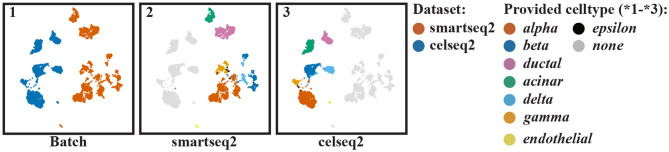
UMAP visualization of the pancreas datasets when no alignment of the cells is performed: (1) colored according to batches, (2) colored according to the clusters found in batch one, and (3) colored according to the clusters found in batch two. The provided cell types by the authors are listed on the right side.

We started by aligning two scRNA-seq datasets of the human pancreas, one measured using Smart-seq2 and the other measured using Cel-Seq2 (Table 1). After selecting genes measured in both batches, normalization and selection of highly variable genes (Methods), we select the top 50 PCs to represent each cell based on the scree plot (Supplementary Figure 2). Panel *batch* of Figure 2 represents the unaligned cells of the two batches, showing that there are two batches.

Next, we clustered the cells in each batch separately. The results are shown in the panels *smartseq*2 and *celseq*2 of Figure 2. Here, we can see that clusters are differently spread, i.e., the clusters in the *celseq*2 data are more heterogeneous compared to the *smartseq*2 data. When aligning the batches, we aim to align these clusters, but at the same time also retain the distribution of cells in the original batches as much as possible.

To guide the alignment by the clustering in the original batches, we automatically matched the clusters between batches (Methods). Supplementary Figure 3 shows which pairs of cells across the two batches are in matching clusters as represented by matrix *M* (Methods), where *M*_*pq*_ > 0 if cells *p* and *q* from the two different batches are in matching clusters and *M*_*pq*_ = 0 otherwise. When aligning the two batches, we aim to move pairs of cells from different batches but in matching clusters close together.

**Figure 3:**
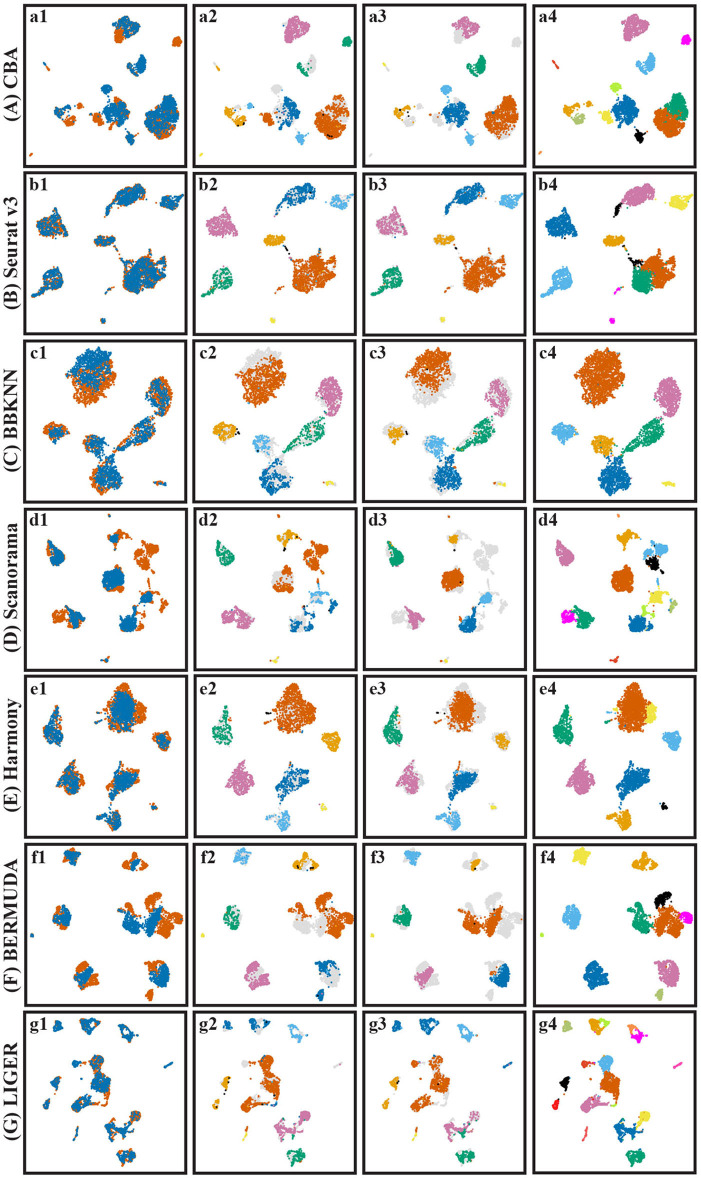
UMAP visualization of different batch effect removal methods for the pancreas datasets. **(A–G)** Show the different alignment methods: **(A)** CBA, **(B)** Seurat v3, **(C)** BBKNN, **(D)** Scanorama, **(E)** Harmony, **(F)** BERMUDA, and **(G)** LIGER. For each panel four color codings are shown again: 1) colored according to the batches, 2) colored according to provided clusters in batch one, 3) colored according to provided clusters in batch two, and 4) colored according to Louvain clusters. The provided cell types listed in Figure 2 are only used to evaluate the resulting alignments and not used in the whole aligning process (including the unsupervised clustering in CBA). In *4), the colors of cells are not corresponding with other subfigures since they are obtained by unsupervised clustering, their colors only indicate the distinction of Louvain clusters.

Based on the PC representation of the cells, the clustering of the different batches, and the information about the matching clusters, we aligned the two pancreas datasets using CBA (Methods). CBA is an autoencoder and therefore finds an embedding space in which the two batches are aligned. Figure 3a1 shows the aligned cells in the embedded space colored according to their batch. The batch effect is removed by CBA as cells from different batches are overlapping. Moreover, from Figure 3a2 (cells in batch one colored according to their clustering) and Figure 3a3 (cells in batch two colored according to their clustering), we do observe that CBA preserves the original clusters (they are not scattered around), and that clusters from different batches with the same annotations end up close to each other.

We compared the performance of CBA with six existing alignment methods: Seurat v3 (Stuart et al., 2019), BBKNN (Polanski et al., 2020), Scanorama (Hie et al., 2019), Harmony (Korsunsky et al., 2019), LIGER (Welch et al., 2019), and BERMUDA (Wang et al., 2019). Figure 3 shows the resulting alignments. From Figure 3b1, it shows that Seurat nicely aligns the batches, which is not the case for Scanorama (Figure 3d1). For the latter one, you can see clusters of aligned data consisting of cells of only one batch, often relating to an original cluster in the separate batches (Figures 3d2,d3). BBKNN (Figure 3c1) also can align both batches but seems to increase the internal variation within the clusters, resulting in touching/overlapping clusters. LIGER (Figure 3g1) also performs well; however, it merges some *acinar* cells with *ductal* cells. Interestingly, BERMUDA, which is also an auto-encoder based alignment network like ours, has more problems in aligning the batches.

Although Seurat seems to align both batches better than the other competing methods, zooming in on the initial clustering of the cells (per batch) shows that Seurat loses some diversity in the individual cell types. That is, all resulting clusters are condensed, not showing any structure within each cluster. In other words, Seurat seems to “over-align” cells, i.e., merging dissimilar cells together and decreasing the cellular diversity, this will be explained in section 3.2.

To quantify the batch alignment performances, we evaluated the resulting alignments using several metrics (Methods) whose results are shown in Table 2. CBA performs competitively across all metrics except for the kBET score, which measures the mixing of the two datasets. Here CBA gets a lower KBET score because it tries to preserve the clustering in the individual datasets. In contrast, Seurat v3 has a high kBET score, and thus mixing the two datasets, but perhaps at the expense of aligning also different clusters in the individual datasets. BBKNN performs best on the metrics that compare the clustering of the aligned data to the clustering in the original datasets, i.e., it best preserved the original clusters, but at the expense of not really aligning the dataset. Harmony does well on all metrics, especially on how well the aligned dataset clusters (SC), but visually the aligned data is not convincing in Figures 3b1,e1.

**Table 2:** Quantitative evaluation of different batch effect removal methods for the pancreas datasets, including kBET^a^, SC^b^, NMI^c^, ARI^d^, and FMI^*e*^.

**Metric**	**CBA**	**Seurat v3**	**BBKNN**	**Scanorama**	**Harmony**	**LIGER**	**BERMUDA**
kBET	0.97	**0.01**	0.73	0.44	0.36	0.84	0.68
SC	0.65	0.61	0.60	0.65	**0.69**	0.62	0.57
NMI	0.80	0.83	**0.91**	0.78	0.89	0.69	0.81
ARI	0.65	0.69	**0.95**	0.58	0.85	0.43	0.60
FMI	0.74	0.77	**0.96**	0.69	0.89	0.57	0.70

a *k-nearest Neighbor Batch Effect Test (Rejection Rate)*.

b *Silhouette coefficient*.

c *Normalized mutual information*.

d *Adjusted Rand Index*.

e *Fowlkes-Mallows Index. Bold values correspond to the best performance (the lowest for kBET and the highest ones for others)*.

### 3.2. CBA Preserves Separation of Cells

**Figure 4:**
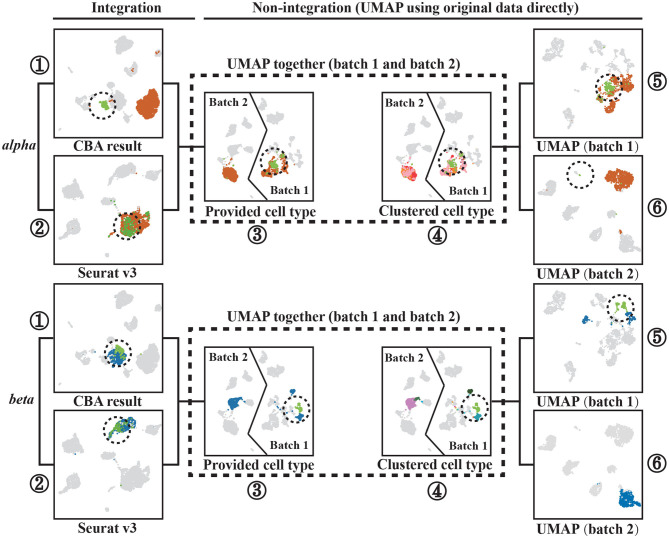
Two cell types, *alpha* and *beta* cells, tracked through different alignment spaces, i.e., UMAPs of: (1) the CBA alignment, (2) the Seurat v3 alignment, (3) and (4) the two batches together but with unaligned cells, (5) only cells in batch one, and (6) only cells in batch two. *Alpha* or *beta* cells are colored the remaining cells are gray colored cells. Encircled Alpha cells are colored with light green to track them in all plots. Separated *beta* cells in batch one are also circled and colored with light green to track them in all plots. Notably, cells are colored according to provided cell types in all plots except (4), these cell types are corresponding with Figure 2 and they are not used when clustering in CBA. In (4), cells are colored according to how they are clustered in the original dataset (using the initial Louvain clustering).

To show that CBA better preserves the local structure of the data, we chose two cell types (*alpha* and *beta* cells) and further analyzed their alignment using CBA and Seurat, with respect to their observed diversity in the original space (Figure 4).

**Figure 5:**
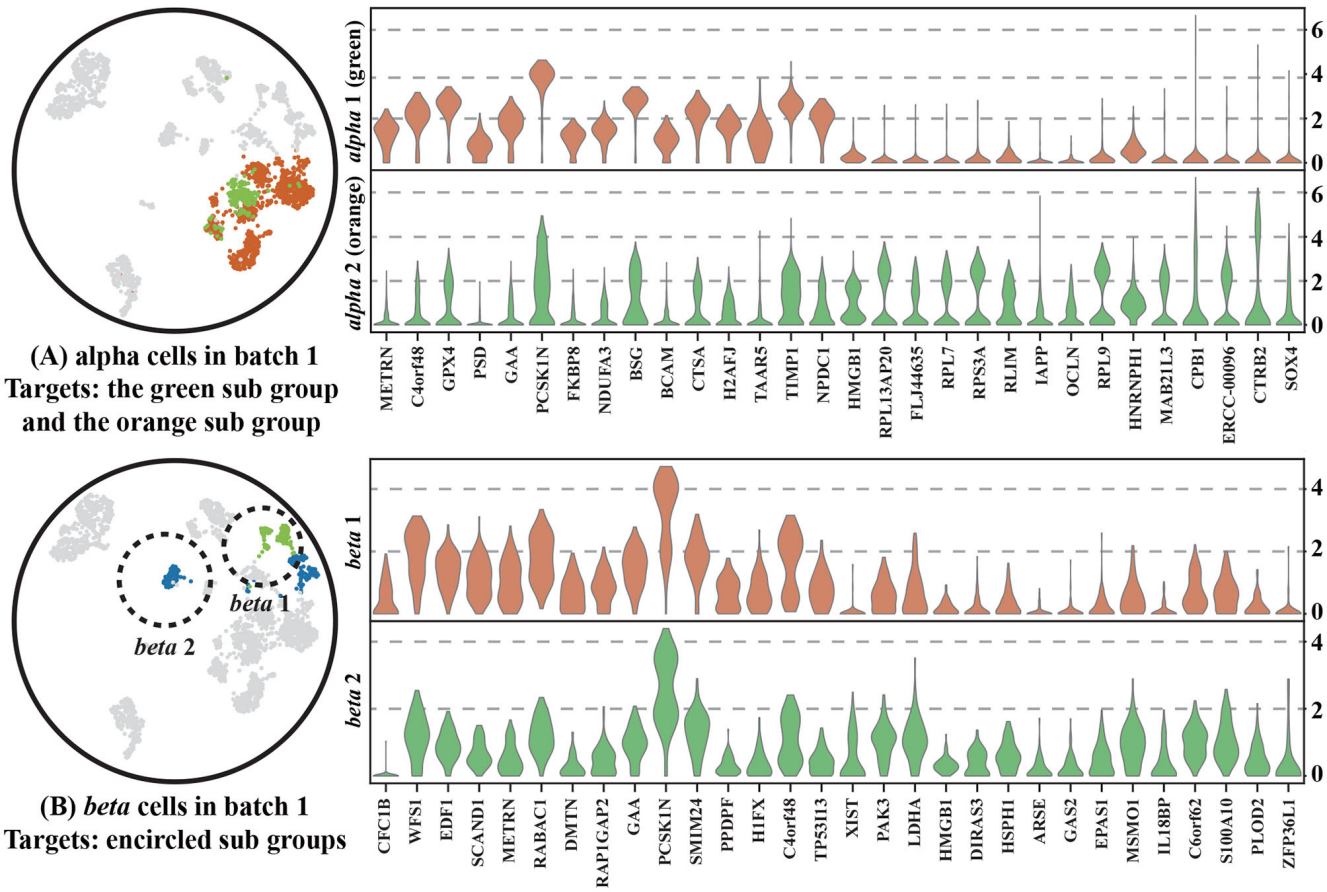
Two violin plots of **(A)** *alpha* cells and **(B)** *beta* cells in Batch 1 showing the expression of the top 30 differential expressed genes between two sub groups within either the *alpha* cells and *beta* cells. Cells within the two sub groups are colored differently and shown on the left of plots.

For the alpha cells, CBA splits the cells in two groups, whereas Seurat merges all alpha cells into one cluster. The cells of one of the groups are marked (encircled, different color) and traced in the other visualizations. We found these cells to be only present in Batch 1, in which they also form a subcluster within *alpha* cells. CBA is able to preserve this separation when aligning the cells. Differential expression analysis between these cells and other *alpha* cells in Batch 1 identified 1,992 deferentially expressed genes (Figure 5A). Some of these top differentially expressed genes have been previously linked to the pancreas or alhpa cells, pinpointing their biological relevance. For example, *PCSK1N* is known to be expressed in alpha cells and transgenic mice overexpressing *PCSK1N* have an obese phenotype (Wei et al., 2004). Mutations in *CPB1* are associated with pancreatic cancer (Tamura et al., 2018). The *CTRB1*-*CTRB2* locus is identified to modify the risk for alcoholic and non-alcoholic chronic pancreatitis (Rosendahl et al., 2018). Animal studies also indicate that *GPX4* plays a major role in inhibiting ferroptosis under multiple conditions (Dai et al., 2020). Although, Seurat merges the cells in the same cluster, we do see that also Seurat picks up these cells and locates them together at the border of the cluster, but not separated as in the CBA result. Hence, CBA preserves the biological information of the cells more prominently.

It is important to note that a separation in one of the batches is not always preserved. For example, in the *beta* cell cluster, CBA regarded that the separation in batch one not strong enough to split cells in the alignment and, similar to Seurat, decides to align all *beta* cells into one cluster. We used *scanpy.tl.rank_genes_groups()* to select 30 differential genes that separate the two *beta* groups and show the distribution of their gene expression levels for both groups. The violin plot (Figure 5B) shows that although these two groups are separated in the raw space, their gene expressions show similar patterns, indicating that these cells are not so different from each other.

### 3.3. CBA Is Not Sensitive to Missing Cell Types

**Figure 6:**
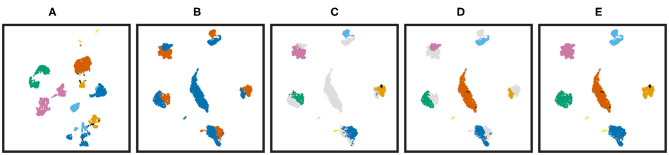
UMAP visualization of imbalanced CBA for the pancreas datasets. *alpha* cells are removed from *smartseq*2 and they are present in *celseq*2. **(A)** The UMAP visualization of both batches when no alignment of the cells is performed and cells are colored according to batches. **(B–E)** Show the CBA performance: **(B)** colored according to batches, **(C)** colored according to the clusters found in batch one, **(D)** colored according to the clusters found in batch two, **(E)** colored according to the clusters found in both batches. The cluster colors are corresponding with Figure 2.

A major motivation behind CBA is the cluster guidance of the alignment. This step depends on the cluster matching between the two datasets. We therefore set out to test how sensitive CBA is to this matching step. Hereto, we removed the *alpha* cells from one the two pancreas datasets. As such, seen from the other dataset, these cells are missing (i.e., there is no matching cluster). Figure 6A shows the UMAP visualization before integration and Figures 6B–E show the resulting CBA alignment. Although *alpha* cells were removed from the *smartseq*2 dataset, both datasets are well-aligned and the *alpha* cells in the *celseq*2 dataset do not match with other clusters in the *smartseq*2 dataset (the matching matrix *M* is shown in Supplementary Figure 4), so missing *alpha* cells from *smartseq*2 will not results in wrongly cluster matching. Moreover, the autoencoder structure and L_*r*_ in our loss function can extract high-level cell features existing in the latent embedding, that help to find matching cells for the *alpha* cells in *celseq*2 in the *smartseq*2 dataset.

### 3.4. Performance Comparison for Mouse Lung Datasets

**Figure 7:**
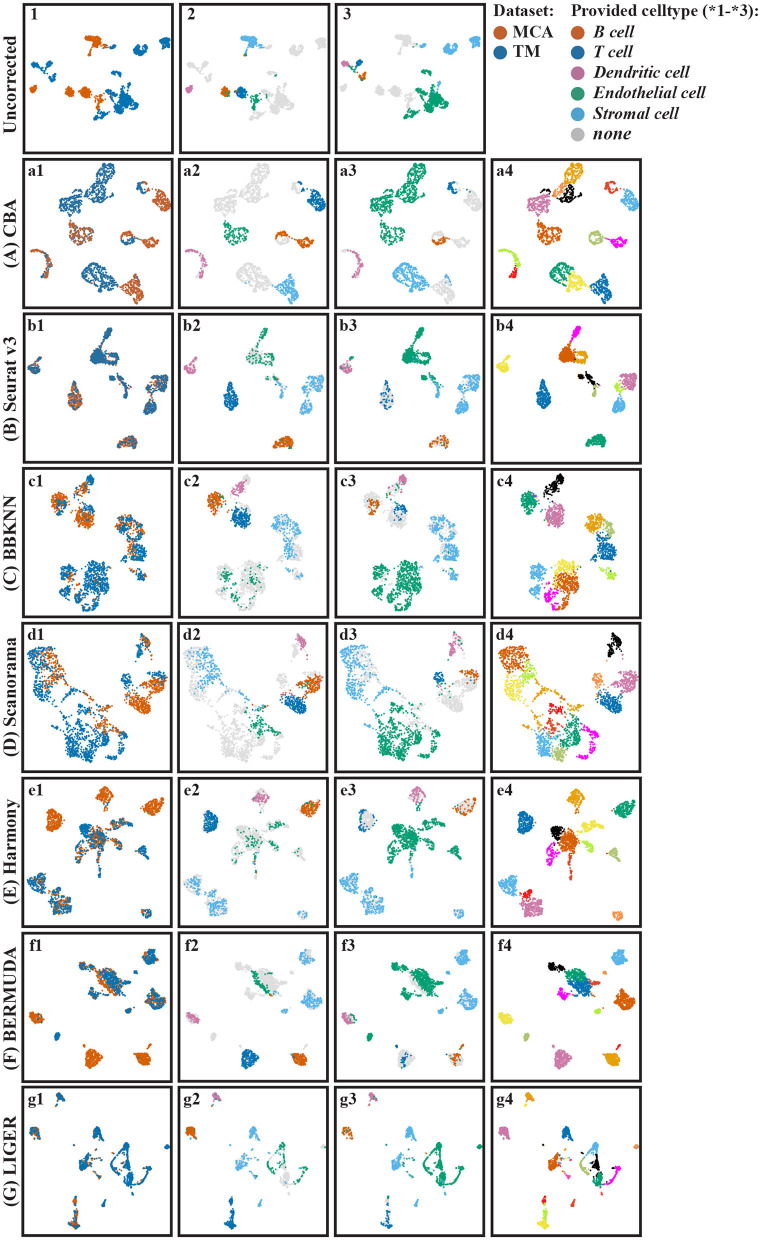
UMAP visualization of different batch effect removal methods for the mouse lung cell datasets. **(A–G)** Show the different alignment methods: **(A)** CBA, **(B)** Seurat v3, **(C)** BBKNN, **(D)** Scanorama, **(E)** Harmony, **(F)** BERMUDA, and **(G)** LIGER. For each panel four color codings are shown again: 1) colored according to the batches, 2) colored according to provided clusters in batch one, 3) colored according to provided clusters in batch two, and 4) colored according to Louvain clusters.

To show that CBA generalizes to other datasets, we aligned two lung datasets from MCA and TM (Table 1). Our results (Figure 7A) show that the batch effects are removed by CBA. Further, we compared the performance of CBA with the other alignment methods (Figures 7B–G). Zooming in on the endothelial cells highlights CBA's ability to merge cells across batches while retaining biological variability. Endothelial cells seems to show high variability in batch2 (Figure 7a3) compared to batch 1 (Figure 7a2). CBA is able to align endothelial cells from batch 1 and batch 2 in the lower part of the integrated data (Figure 7b3), while preserving the diversity of batch 1. On the other hand, Seurat, BBKNN, Harmony, and LIGER merge all cells in one group, while BERMUDA and Scanorama are not able to merge cell from the two batches (i.e., they are still separable in the integrated embeddings). The quantitative comparison between the methods (Supplementary Table 1) shows a similar picture as for the pancreas datasets: CBA has a competitive performance, especially for the clustering measures, although it again has a low kBET score because it preserves the cluster structure in the original batches.

## 4. Discussion

With the development of single cell RNA sequencing technologies, an increasing number of cell datasets are sampled by multiple platforms with different experimental settings. While current methods can generally resolve batch differences across scRNA-seq datasets, local batch-specific structures and separations of cells are not fully preserved. We developed CBA, a cluster-guided batch alignment for single cell RNA sequencing. Besides matching similar cells across batches, CBA maintains the local data structure in the separate batches as much as possible. A carefully designed auto-encoder is used to embed cells from two batches into the same space. To achieve our expectation, two different cell-cell distances are preserved: (1) cell-cell distances within the same cluster of cells in a single batch, and (2) cell-cell distances within matched cell clusters across different batches. The cluster guidance is fully unsupervised as the initial clusters are detected from each batch using Louvain clustering. The training of CBA is fast without requiring too much memory.

Our results show that CBA is able to integrate cells from two batches across different datasets. Compared to other single cell alignment methods (*Seurat v3, BBKNN, Scanorama, Harmony, LIGER*, and *BERMUDA*), CBA avoids “over-aligning” batches, i.e., preserving the separation of clusters in both original batches. Compared with CBA, other cell alignment procedures tend to merge these batch-specific clusters during the alignment. We have shown that the preserved separation by CBA of dissimilar cells in a single batch is indeed reflected by biologically-relevant deferentially expressed genes between the preserved batch-specific clusters. Moreover, CBA is not sensitive to missing matching clusters. It is important to note that CBA aligns pairs of datasets and recursive alignment is needed if more than one pair of batches should be aligned. In addition, we have only shown that CBA can be used to integrate data from the same species.

With our cluster-guided batch alignment (CBA) framework we have shown the potential of a structure preserving alignment procedure when matching two single cell RNAseq datasets. Because of its versatility it is interesting to explore such an alignment procedure for different data types, like for example ATACseq data. Also, it is interesting to explore additional structure preserving constraints during alignment. Formulating the alignment into an autoencoder framework turned out to be very flexible because additional constraints can easily be added as additional loss functions.

## Data Availability Statement

The implementation of CBA is available at: https://github.com/GEOBIOywb/CBA.git. The datasets analyzed for this study can be found at: https://doi.org/10.5281/zenodo.4528994.

## Author Contributions

WY developed the method, performed the experiments, interpreted the results, and drafted the manuscript. AM and MR guided the method development, designed the experiments, interpreted the results, and wrote the manuscript. All authors contributed to the article and approved the submitted version.

## Conflict of Interest

The authors declare that the research was conducted in the absence of any commercial or financial relationships that could be construed as a potential conflict of interest.

## Footnotes

^1^https://satijalab.org/seurat/pancreas_integration_label_transfer.html

^2^https://figshare.com/s/865e694ad06d5857db4b

^3^https://figshare.com/projects/Tabula_Muris_Transcriptomic_characterization_of_20_organs_and_tissues_from_Mus_musculus_at_single_cell_resolution/27733
