# Supplementary material for: CBA: Cluster-Guided Batch Alignment for Single Cell RNA-seq
Source: Front Genet. 2021 Apr 13;12:644211. doi: 10.3389/fgene.2021.644211 (PMC8076908; doi:10.3389/fgene.2021.644211)
Supplement: Supplementary file 1 [file Data_Sheet_1.PDF]

Supplementary Material

1 SUPPLEMENTARY TABLES AND FIGURES

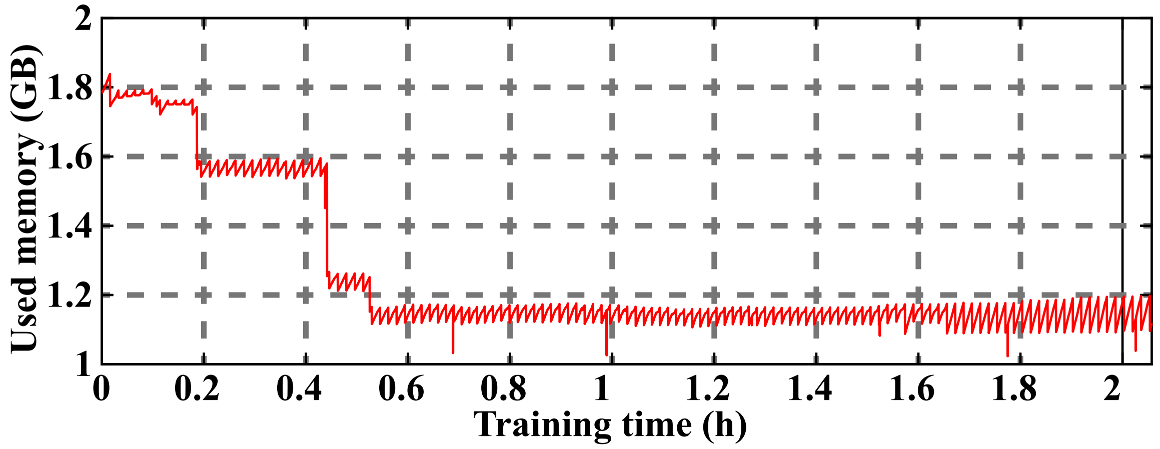

Figure S1. Used memory versus the training time, totally ten thousand epochs are shown in this figure. The whole training time was 2.06 hours, so the training time per epoch was approximately 1s. The showed memory is the resident set size (RSS) memory occupied by the process, which is held in RAM (main memory). The line drops because of the release of some memory.

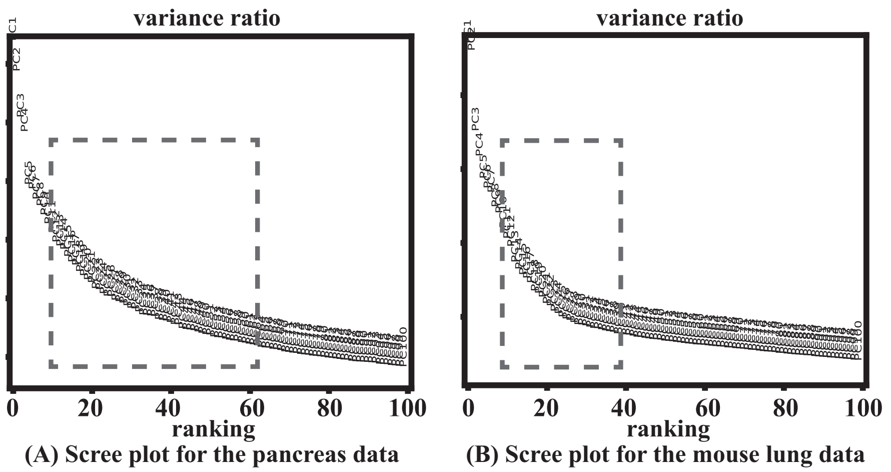

Figure S2. Scree plots for (A) the pancreas datasets and (B) the mouse lung datasets. Both plots reflect the contribution of single PCs generated by PCA, we decide the number of PCs based on the bending point of the variance explained per component.

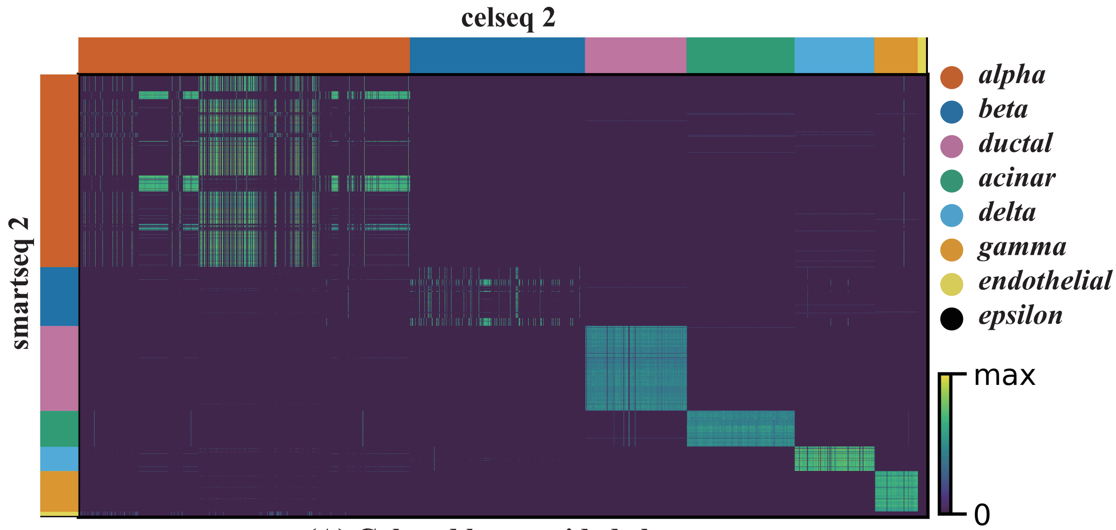

(A) Colored by provided clusters

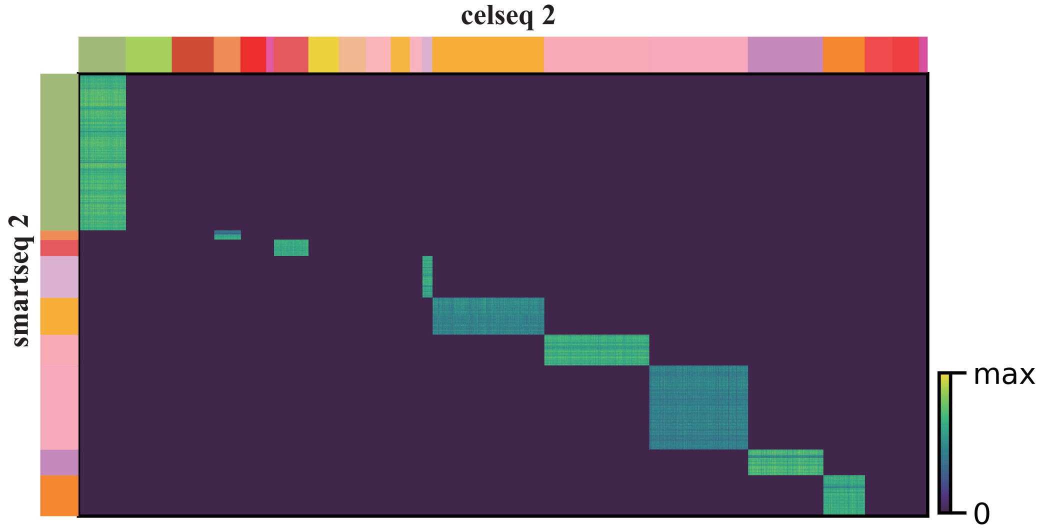

(B) Colored by our clusters by Louvain

Figure S3. Matrix M for the pancreas datasets which indicates whether a cell in batch 1 (rows) belongs to a cluster that matches with the cluster belonging to a cell in batch 2. (A) Cells from both batches are sorted by their respective given clusters, colors at the top/on the left of the plot represent the different provided clusters by their authors in each of the two batches, respectively. (B) Cells from both batches are sorted by our clusters and colors are not matched with given clusters.

Table S1. Quantitative evaluation of different batch effect removal methods for the mouse lung cell datasets, see Table 2 for further description of this table

Metric	CBA	Seurat v3	BBKNN	Scanorama	Harmony	LIGER	BERMUDA
kBET	0.35	**0.01**	0.54	0.70	0.48	0.96	0.86
SC	**0.82**	0.60	0.62	0.60	0.67	0.67	0.43
NMI	**0.71**	0.69	0.70	0.60	0.656	0.62	0.68
ARI	0.37	**0.46**	0.43	0.32	0.39	0.31	0.42
FMI	0.54	**0.61**	0.58	0.49	0.55	0.48	0.57

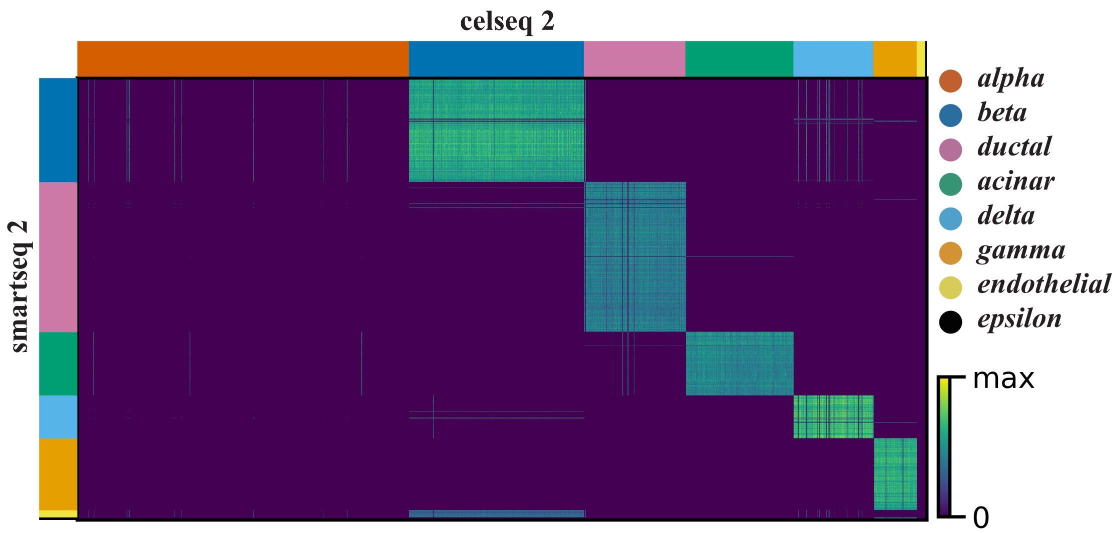

Figure S4. Matrix M for the pancreas datasets when $alpha$ cells are removed from $smartseq2$. Cells from both batches are sorted by their respective provided clusters, colors at the top/on the left of the plot represent the different provided clusters by their authors in each of the two batches, respectively.
